# Fatigue Crack Propagation of Corroded High-Strength Steel Wires Using the XFEM and the EIFS

**DOI:** 10.3390/ma16134738

**Published:** 2023-06-30

**Authors:** Jianchao Zhu, Zhiyu Jie, Chao Chen, Hao Zheng, Weiguo Wang

**Affiliations:** 1 Ningbo Communication Construction Engineering Testing Center Co., Ltd., Ningbo 315124, China; 2 Department of Civil Engineering, Ningbo University, Ningbo 315211, China

**Keywords:** fatigue crack propagation, high-strength steel wires, corrosion pits, extended finite element method, equivalent initial flaw size

## Abstract

A fatigue test and numerical simulation on corroded high-strength steel wires with multiple corrosion pits were conducted. A new approach combining the eXtended Finite Element Method (XFEM) and the Equivalent Initial Flaw Size (EIFS) was proposed to investigate three-dimensional fatigue crack growth and life prediction. The EIFS values for the steel wires were determined under various stress ranges and corrosion pit conditions. The fatigue crack propagation path, the fatigue life, and the stress variation under different pit types and depths were investigated. The results reveal a significant linear relationship between the maximum principal stress range and the fatigue life in logarithmic coordinates for steel wires with various pit types. Additionally, the EIFS is found to be dependent on the stress range and the pit depth. All the predicted outcomes fall within a range of twice the margin of error. The accuracy of this novel method is further verified by comparing predicted results with the test data. This research contributes to a better understanding of the fatigue performance of corroded high-strength steel wires and can assist in the design and maintenance of notched components.

## 1. Introduction

In recent years, with the rapid development of the economy, there has been a significant increase in transportation demand. Cable-supported bridges are becoming increasingly popular among designers worldwide due to their reasonable structural system, powerful spanning ability, strong practicality, and economy. Consequently, the number of cable-supported bridges around the world is increasing day by day. When the PE sheath is damaged, the galvanized parallel steel wires within the cable are subjected to the combined effects of alternating loads and corrosive factors. The steel wire is susceptible to corrosion damage, and pitting is the most common and dangerous form of localized corrosion. Pit corrosion can result in a reduction in the fatigue strength and life of the steel wire, eventually leading to wire fracture [1]. In practice, the service life of cables in various countries is usually less than 20 years. Corrosion pits have become an important disease that endangers the operational safety of cable-supported bridges.

Many methods have been proposed for the fatigue assessment of corroded high-strength steel wires, such as the stress method, the energy method and the fracture mechanics method, etc. Jie et al. [2,3,4] used the stress method and the energy method for the fatigue life prediction of pitted/cracked high-strength steel wires, and the effectiveness and accuracy were validated by test results. Bai et al. [5] proposed a multiparameter Weibull probability model to describe the quantitative influences of corrosion on the fatigue life of high-strength steel wires. Xue et al. [6] provided a fatigue crack growth formula considering the coupling effect of corrosion and fatigue with different corrosion degrees. When a corroded steel wire is subjected to fatigue loading, cracks often originate from corrosion pits [7]. It assumes that high-strength steel wires generate cracks from pits, and fracture mechanics is conveniently employed to investigate fatigue crack growth and life prediction. Generally, the fatigue life is determined by the initial crack size, the critical crack size, and the crack growth rate [8]. However, it is hard to obtain the initial crack size accurately due to the presence of pitting. A newly developed Equivalent Initial Flaw Size (EIFS) methodology [9] is discussed for crack growth-based fatigue life prediction. The EIFS methodology is based on the Kitagawa–Takahashi diagram [10] and the El Haddad et al. model [11]. The fatigue life of specimens can be predicted by growing the crack from the EIFS to the final failure. Zhang et al. [12] proposed an improved EIFS for corrosion fatigue life prediction, and it assumed that pitting corrosion and small crack growth were equivalent to a part of the long crack growth process in the corrosion fatigue process. Based on the updated Kitagawa–Takahashi diagram, Lian et al. [13] gave a normalized EIFS method considering the small crack growth behavior, and theoretical predictions showed good agreement compared with the experimental results. Sun et al. [14] developed a novel corrosion fatigue prediction approach for corroded RC beams based on the EIFS, and two methods were used to derive the EIFS in the presence of corrosion: K-T diagram method and calibrating to test data method. Wang et al. [15] applied the EIFS method to estimate the fatigue life of corroded steel wires, and this method was verified by experimental results in the open literature. In corroded steel wires, multiple corrosion pits have also been widely observed, so their interaction effects on fatigue behavior are required to be considered in the flaw assessment [16].

In general, there are several methods for simulating crack propagation: the finite element method, the boundary element method, the meshless method, the finite difference method, etc. At present, a lot of research has been carried out on the finite element simulation of fatigue crack propagation for high-strength steel wires. However, it is necessary to continuously adjust elements and meshes to accommodate discontinuous interfaces for fatigue crack growth. Thus, the Extended Finite Element Method (XFEM) has been proposed as a powerful numerical technique for modeling problems with discontinuous displacement fields. One of the advantages of XFEM is that it can effectively handle discontinuities without the need to remesh the computational domain, which is a significant advantage over the traditional finite element method. Nikfam et al. [17] validated the fatigue crack growth procedure against constant amplitude HCF experiments on notched steel plates from other researchers by using the XFEM. Jie et al. [18] used the XFEM applied to estimate the fatigue life of unrepaired and CFRP patch-repaired cracked welded joints. Zeng et al. [19] established a numerical model based on the XFEM to analyze the cracking of the diaphragm of an experimental 1:2 scaled deck segment model. Chen et al. [20] investigated the effects of residual stresses on crack propagation under different loading conditions using the XFEM combined with Paris’ law. Jiang and Wang [21] studied the stress intensity factors at crack tips and dynamic fatigue crack propagation at the U rib–diaphragm connection by the XFEM, and the difference in fatigue crack propagation life and cumulative energy release rate under different corrosion levels were investigated. Up until now, there have been few studies using the XFEM for the fatigue assessment of corroded high-strength steel wires.

In this paper, a novel approach combining the XFEM and the EIFS based on the finite element software ABAQUS was proposed to investigate the three-dimensional fatigue crack propagation of high-strength steel wires with multiple pits. The basic principle of the XFEM and the concept of the EIFS were introduced. To validate the proposed approach, fatigue tests were conducted on high-strength steel wires with multiple pits under constant amplitude loading. The EIFS values of steel wires were calculated for different stress ranges and corrosion pits. The fatigue crack propagation path, the fatigue life, and the stress variation were investigated under different pit types and depths. The simulation results were then compared with the experimental data obtained from fatigue tests to assess the accuracy and reliability of the numerical simulations. 

## 2. Basic Knowledge

### 2.1. Extended Finite Element Method (XFEM)

The eXtended Finite Element Method (XFEM) has emerged as an effective solution for handling discontinuous problems, particularly in the context of crack growth analysis. In comparison to the traditional finite element analysis method, it has many advantages, such as: the mesh being relatively independent of the crack, the absence of singular elements when dividing the mesh, and the elimination of the need for remeshing, etc. 

**Figure 1:**
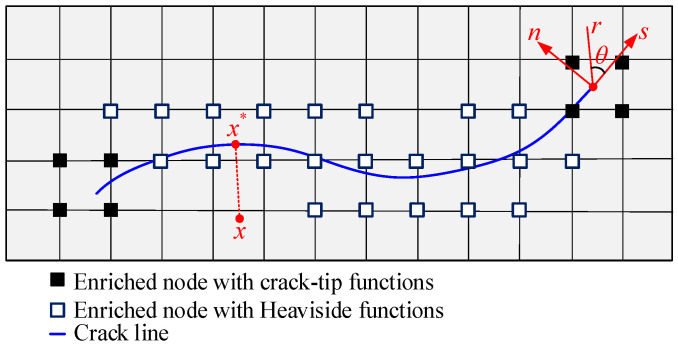
An enriched domain for a crack and a polar coordinate system.

Based on the displacement function of the conventional finite element method, an enrichment function that can reflect the displacement discontinuity of the crack surface and the displacement of the crack tip is added to improve the calculation efficiency. The expression of the displacement function is written as follows:

(1) $$u\left(x\right)=\sum_{i\in N}N_{i}\left(x\right)u_{i}+\sum_{i\in N_{\Gamma }}N_{i}\left(x\right)H\left(x\right)a_{i}+\sum_{i\in N_{\Lambda }}N_{i}\left(x\right)\sum_{\alpha =1}^{m}F_{\alpha }\left(x\right)b_{i}^{\alpha }$$

where $N_{i}\left(x\right)$ is the shape function, $u_{i}$ is the classical nodal displacement, $a_{i}$ and $b_{i}^{\alpha }$ are the additional degrees of freedom of the node, *m* is the number of enriched functions at the crack tip, *N* is the normal node set, $N_{\Gamma }$ is the node set of the element penetrated by the crack, $N_{\Lambda }$ is the node set of the element including the crack tip, and H(x) is the jump function. The discontinuous jump function is given as:

(2) $$H\left(x\right)=\left\{ \begin{array}{l} 1\;\mathrm{if}\;\left(x-x^{*}\right)n\geq 0\\ -1\;\mathrm{otherwise} \end{array}\right.$$

where *x* is an arbitrary point near the crack face, $x^{*}$ is the closest point to *x* located on the crack face (Figure 1), *n* is the outward unit vector normal to the crack at $x^{*}$. The crack tip enrichment function $F_{\alpha }\left(x\right)$ is expressed as follows:

(3) $$F_{\alpha }\left(x\right)=\left[\sqrt{r}\sin\left(\frac{\theta }{2}\right),\sqrt{r}\cos\left(\frac{\theta }{2}\right),\sqrt{r}\sin\left(\theta \right)\sin\left(\frac{\theta }{2}\right),\sqrt{r}\sin\left(\theta \right)\cos\left(\frac{\theta }{2}\right)\right]$$

where *r* and 𝜃 are the polar coordinates in the local crack tip (Figure 1).

### 2.2. Equivalent Initial Flaw Size (EIFS) Theory

The fatigue life of a component can be divided into two distinct phases: crack initiation life and crack growth life. By utilizing an equivalent conversion method, the crack initiation life can be considered a portion of the crack propagation life. Therefore, it assumes the existence of an Equivalent Initial Flaw Size (EIFS) $a_{\mathrm{EIFS}}$. Fatigue crack propagates from $a_{\mathrm{EIFS}}$ to the critical flaw size $a_{c}$. Total fatigue life can be defined mathematically as:

(4) $$N=N_{i}+N_{p}==\int_{a_{\mathrm{EIFS}}}^{a_{c}}\frac{1}{\left(\frac{da}{dN}\right)}da$$

(5) $$\frac{da}{dN}=C{\left(\Delta K\right)}^{m}=C{\left(Y\Delta \sigma \sqrt{\pi a}\right)}^{m}$$

where $N_{i}$ is the fatigue initial life, $N_{p}$ is the fatigue growth life, d*a*/d*N* is the crack growth rate, *C* and *m* are empirically material constants, Δ*K* is the stress intensity factor (SIF) range, *Y* is the crack shape correction factor, Δ*σ* is the nominal stress range, and *a* is the crack depth.

**Figure 2:**
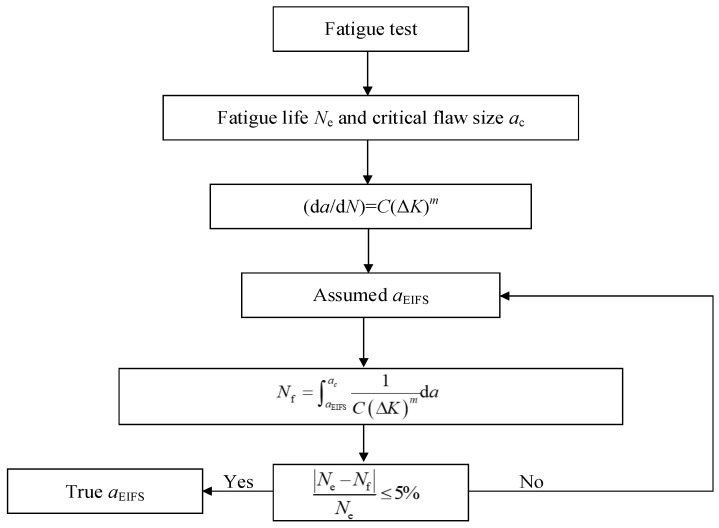
The back extrapolation method used to calculate the EIFS.

The most applicable methods to estimate the EIFS are the back extrapolation method, the Kitagawa–Takahashi diagram method, and the time to crack initiation method [22]. In this context, we will focus on the back extrapolation method, which is typically employed for calculating the EIFS. This approach utilizes fatigue crack growth analysis by assuming an initial crack geometrical size to match the material failure data. The calculation process is illustrated in Figure 2. The relationship between the EIFS and the stress range can be expressed as follows:

(6) $$a_{\mathrm{EIFS}}=k{\left(\Delta \sigma \right)}^{\eta }$$

where *k* and η are material-dependent constants that are determined based on experimental data and calibration, and Δσ is the stress range.

## 3. Experimental Program

**Figure 3:**
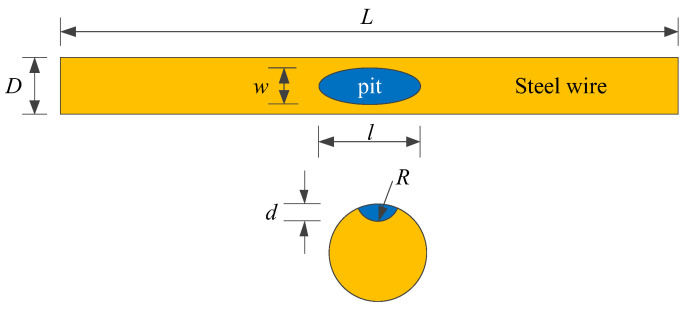
Steel wire with a pit.

**Figure 4:**
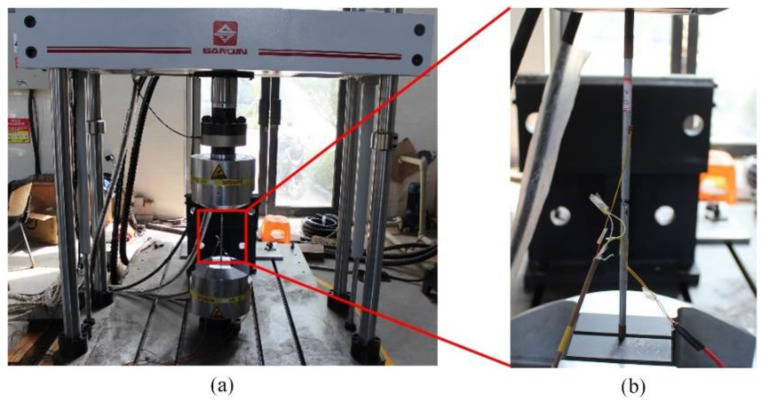
Fatigue test loading setup (**a**) and specimen failure (**b**).

The corrosion behavior of high-strength galvanized steel wires used in bridge structures is subjected to substantial randomness, where the degree of corrosion, location, and size of corrosion pits often exhibit significant uncertainties. Such uncertainties have impeded the study of steel wires, necessitating the development of a more reliable approach. To address this issue, this study focused on high-strength galvanized steel wires with a tensile strength of 1770 MPa and a yield strength of 1580 MPa, where semi-ellipsoid and hemisphere artificial notches were fabricated. The chemical composition of high-strength steel wires can be found in ref. [23]. The steel wire with a pit had a diameter of 7 mm and a length of 500 mm, as shown in Figure 3. The pit types and sizes are presented in Table 1. The fatigue test was performed using an electro-hydraulic servo testing machine with a frequency of 10 Hz and a stress ratio of 0.1. Sine wave loading is applied, with stress ranges of 420 MPa, 480 MPa, 540 MPa, and 600 MPa, as indicated in Table 2. Figure 4 plots the fatigue test loading setup and specimen failure.

## 4. Results and Discussions

**Figure 5:**
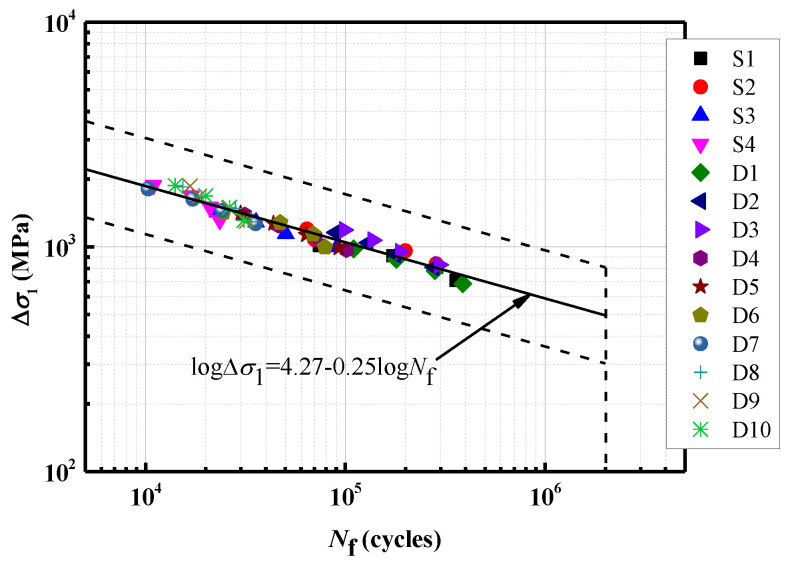
S-N curves for steel wires with multiple pits.

The relationship between the maximum principal stress range and the fatigue life of steel wires with multiple pits is determined through regression analysis using the least squares method, as depicted in Figure 5. The findings reveal that the presence of pits on a steel wire can indeed lead to a decrease in fatigue life. Furthermore, it is observed that deeper pits result in a greater reduction in fatigue life. Among steel wires with various pit depths, the fatigue life is predominantly influenced by deeper pits, while shallow pits have a negligible effect. When plotting the maximum principal stress range against the fatigue life on logarithmic coordinates, a strong linear relationship is observed for steel wires with different pit types. The relationship can be expressed as follows:

(7) $$\log\Delta \sigma_{1}=4.27-0.25\log N_{f}$$

where $\Delta \sigma_{1}$ is the maximum principal stress range, and $N_{f}$ is the fatigue life. This linear relationship provides a convenient means of estimating the fatigue life of steel wires with different pit types based on the maximum principal stress range.

**Figure 6:**
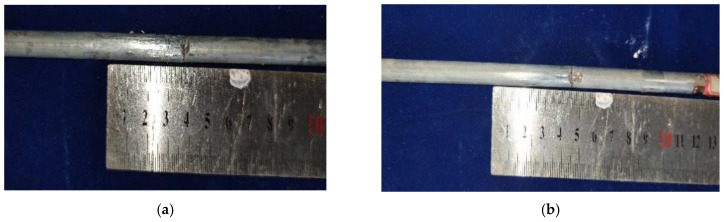
Fatigue crack growth for pitted steel wires. (**a**) a single pit; (**b**) double pits.

**Figure 7:**
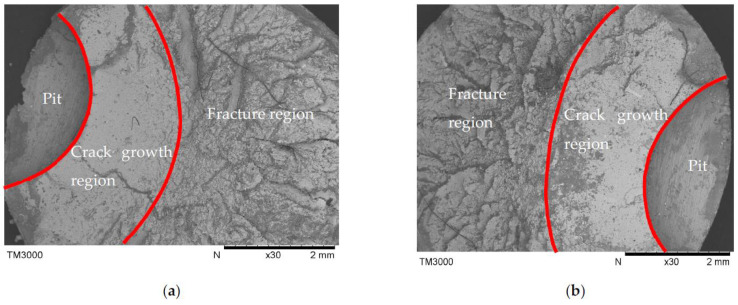
Fatigue fracture morphology for pitted steel wires. (**a**) a single pit; (**b**) double pits.

The fatigue crack growth and fracture morphology of pitted steel wires are illustrated in Figure 6 and Figure 7. These figures provide visual insights into the characteristics of fatigue crack propagation and fracture mechanisms. It can be observed that the fatigue crack propagates transversely in a linear manner along the steel wires. Deeper pits exhibit a higher susceptibility to the formation and propagation of fatigue cracks due to the increased stress concentration caused by these pits. Fatigue cracks typically initiate at the pit mouth or bottom and propagate continuously under cyclic loading conditions. Eventually, a sudden fracture occurs when the cross-section of the wire becomes unable to withstand external loads. 

The fracture surface of the steel wires exhibits distinctive regions of smoothness and roughness. The smooth regions are associated with the initiation and early stages of crack growth, where the crack front remains relatively flat and smooth. As the crack propagates, it encounters various obstacles and interactions, leading to the formation of rough regions on the fracture surface. Overall, the observed fatigue crack growth patterns and fracture morphology highlight the influence of pit depth on crack initiation and propagation, as well as the ultimate failure of the pitted steel wires.

## 5. Fatigue Crack Growth and Life Prediction

### 5.1. EIFS

The critical crack size was determined to be equal to one-third of the diameter of the steel wire. The values of the constants *C* and *m* were found to be 1.8442 × 10^−9^ and 1.3225, respectively, as reported in Ref. [24]. For round steel wires with an elliptical crack subjected to axial tensile loading, the crack shape correction factor *Y* was written as [23]:

(8) $$Y\left(\frac{a}{D}\right)=0.7282-2.1425\left(\frac{a}{D}\right)+18.082{\left(\frac{a}{D}\right)}^{2}-49.385{\left(\frac{a}{D}\right)}^{3}+66.114{\left(\frac{a}{D}\right)}^{4}$$

In the analysis of steel wires with a single pit and double pits, an elliptical crack shape is assumed. The EIFS values for steel wires with a single pit and double pits under different stress ranges are provided in Table 3 and Table 4, respectively, based on the experimental results and the information depicted in Figure 2. It is evident from the tables that the EIFS is influenced by both the stress range and the depth of the pit. With the increase in the stress range and the pit depth, the EIFS increases. Deeper pits have a more pronounced effect on the EIFS compared to shallow pits. This observation aligns with expectations, as higher stress ranges correspond to higher stress levels, which can result in larger crack sizes. Under the same stress range, deeper pits tend to lead to larger crack initiation. Additionally, it is observed that an increase in the stress range of 60 MPa corresponds to an EIFS increase of approximately 0.2 mm. 

### 5.2. Numerical Modelling by the New Approach

**Figure 8:**
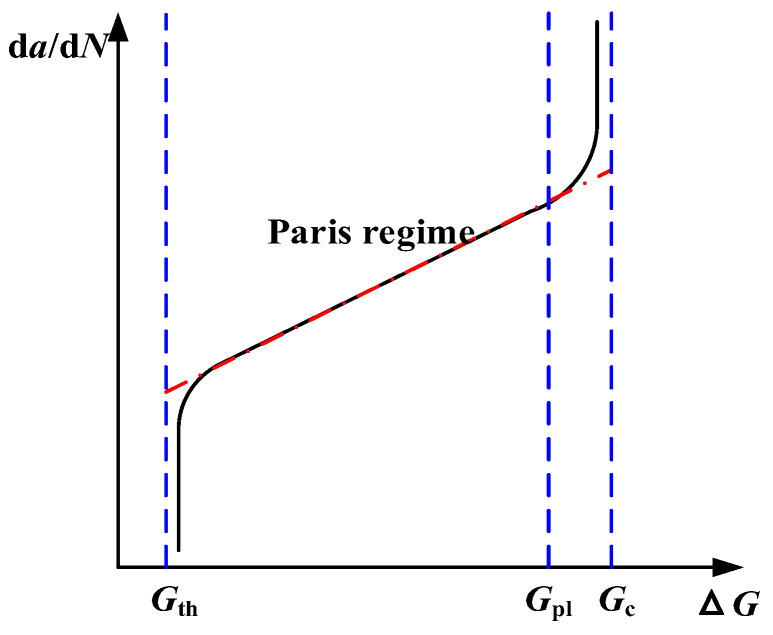
Relationship between fatigue crack growth rate and energy release rate.

A novel methodology integrating the XFEM and the EIFS has been employed to investigate the fatigue crack propagation behavior and predict the life of high-strength steel wires containing multiple pits. The Paris formula is primarily utilized to establish the relationship between the fatigue crack growth rate and the SIF range. The fatigue crack growth rate is depicted based on the energy release rate range Δ*G* in Figure 8. Within this figure, *G*_th_ represents the energy release rate threshold, *G*_pl_ denotes the upper limit of the energy release rate, and *G*_c_ signifies the critical value of the energy release rate.

It needs to be satisfied as the following equation for the fatigue crack initiation [18]:

(9) $$\frac{N}{c_{1}{\left(\Delta G\right)}^{c_{2}}}\geq 1.0$$

where *c*_1_ and *c*_2_ are material parameters of the fatigue crack initiation. The fatigue crack growth rate as a function of the energy release rate range is written as follows:

(10) $$\frac{da}{dN}=c_{3}{\left(\Delta G\right)}^{c_{4}}$$

where *c*_3_ and *c*_4_ are material parameters of the fatigue crack growth. Based on the relationship between Δ*G* and Δ*K*, the expressions of *c*_3_ and *c*_4_ are presented as follows:

(11) $$c_{3}=C{\left(E^{\prime }\right)}^{c_{4}}$$

(12) $$c_{4}=m/2$$

where

(13) $$E^{\prime }=\left\{ \begin{array}{l} E\;\mathrm{Plane}\;\mathrm{stress}\\ \frac{E}{1-\nu^{2}}\;\mathrm{Plane}\;\mathrm{strain} \end{array}\right.$$

where *E* and *ν* are the elastic modulus and Poisson’s ratio, respectively. 

#### 5.2.1. Steel Wires with a Single Pit

**Figure 9:**
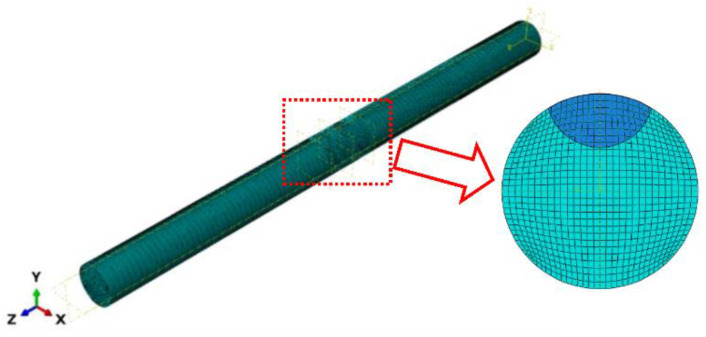
FEM for a steel wire with an EIFS.

The XFEM model of a high-strength steel wire employing the hexahedral element (C3D8R) in the ABAQUS software is shown in Figure 9. The elastic modulus of high-strength steel wires was 200 GPa, with a Poisson’s ratio of 0.3. To enhance the model operation efficiency and result accuracy, the mesh size of 0.2 mm was refined near the notch region. One end of the steel wire was fixed, while a stress range was applied at the other end. The values of parameters *c*_1_ and *c*_2_, which were associated with the fatigue crack initiation criterion, were determined to be 0.5 and −0.1, respectively [25]. Additionally, based on Equations (11) and (12), the values of parameters *c*_3_ and *c*_4_ were equal to 5.9 × 10^−6^, *c*_4_ = 0.66125, respectively. The maximum principal stress damage criterion was chosen for predicting the fatigue crack growth behavior. 

**Figure 10:**
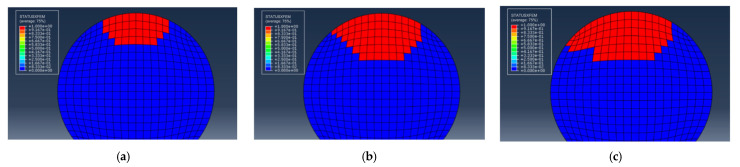
Fatigue crack growth for steel wires with an EIFS. (**a**) *N* = 0; (**b**) *N* = 56,315 cycles; (**c**) *N* = 107,830 cycles.

For the example of selecting S1-3 in the single-pit steel wire, the initial pit depth is 0.5 mm, and the EIFS is 0.6839 mm under the stress range of 540 MPa. The crack shape observed in this scenario is a resemblance to the assumed semi-elliptical shape. Figure 10 plots the fatigue crack growth behavior for steel wires with a single pit. The fracture element is represented by the red color 1, while the unbroken element is depicted by the blue color 0. As the number of cycles to failure increases, the fatigue crack predominantly initiates from the bottom of the pit and gradually propagates toward both ends of the steel wire. 

**Figure 11:**
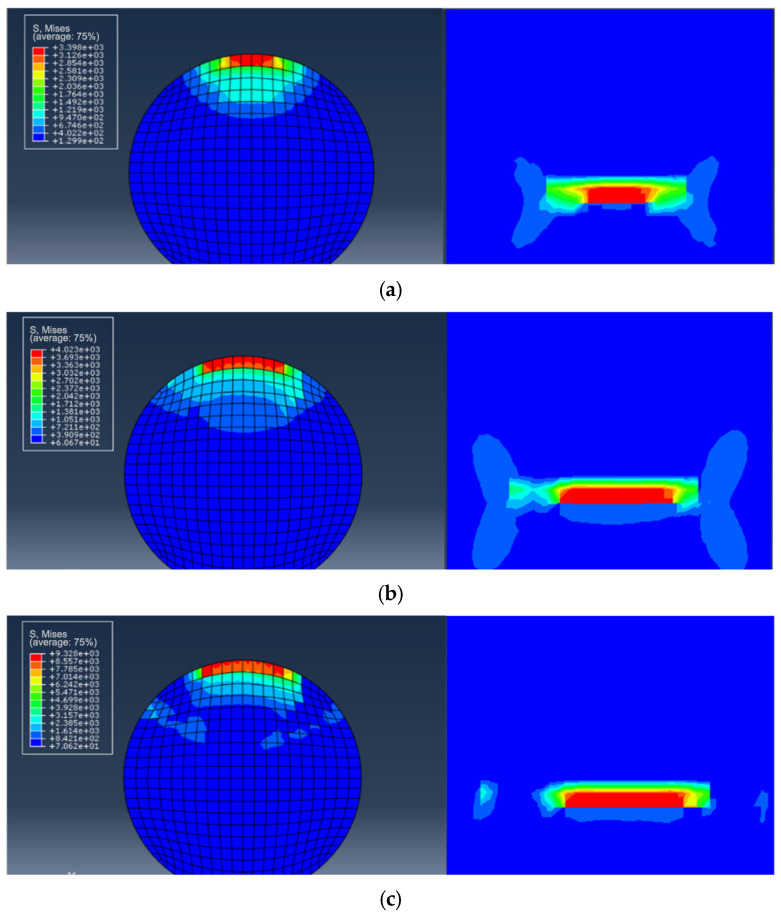
The stress state during fatigue crack propagation for steel wires with an EIFS. (**a**) *N* = 0; (**b**) *N* = 56,315 cycles; (**c**) *N* = 107,830 cycles.

**Figure 12:**
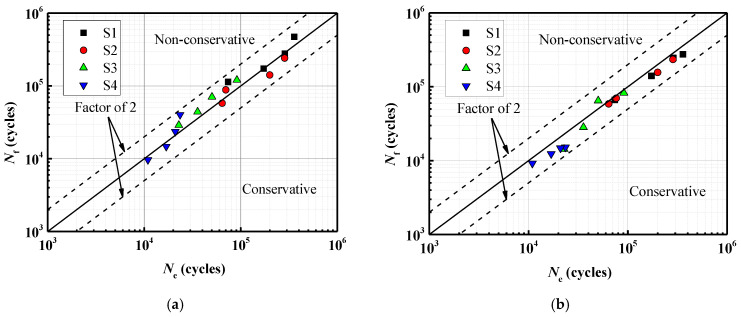
A comparison between fatigue life prediction results and test results for steel wires with an EIFS. (**a**) the maximum principal stress method; (**b**) the new method.

To investigate the stress distribution during the fatigue crack propagation process of the steel wire, the stress variation of the cross-section is presented in Figure 11. It can be observed that the stress state of the steel wire differs at various loading cycles, and the maximum von Mises stress consistently occurs at the crack’s outer surface. With the increase in the cycles to failure, the fatigue crack gradually grows towards the center, indicating an expansion in the stress region. The stress contour of the steel wire transitions from an initial butterfly shape to a localized point, and the stress concentration deepens at the center. Fatigue life prediction results for steel wires with a single pit by using the maximum principal stress method and the new method are shown in Figure 12. It is noteworthy that all predicted results are within a range of twice the bandwidth. The newly proposed method can be successfully used to assess the fatigue life of steel wires with a single pit. 

#### 5.2.2. Steel Wires with Double Pits

**Figure 13:**
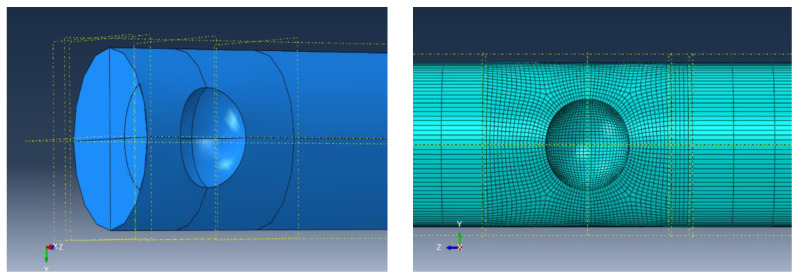
FEM for a steel wire with a pit and an EIFS.

According to the findings reported in ref. [2], it is observed that steel wires with double pits typically exhibit the single largest main crack, with the maximum stress occurring at the bottom of the deeper pit. Consequently, it can be assumed that the deep pit behaves as the main crack, while the shallower pit remains in its original state (Figure 13). The proposed new method is employed to simulate the fatigue crack growth in this scenario. 

**Figure 14:**
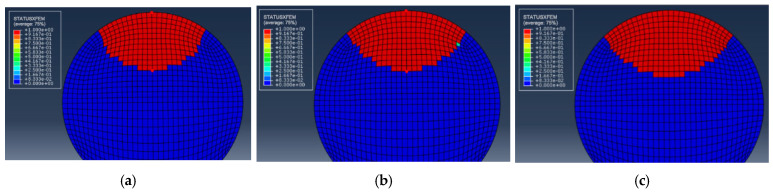
Fatigue crack propagation for steel wires with a pit and an EIFS. (**a**) *N* = 0; (**b**) *N* = 6680 cycles; (**c**) *N* = 12,989 cycles.

**Figure 15:**
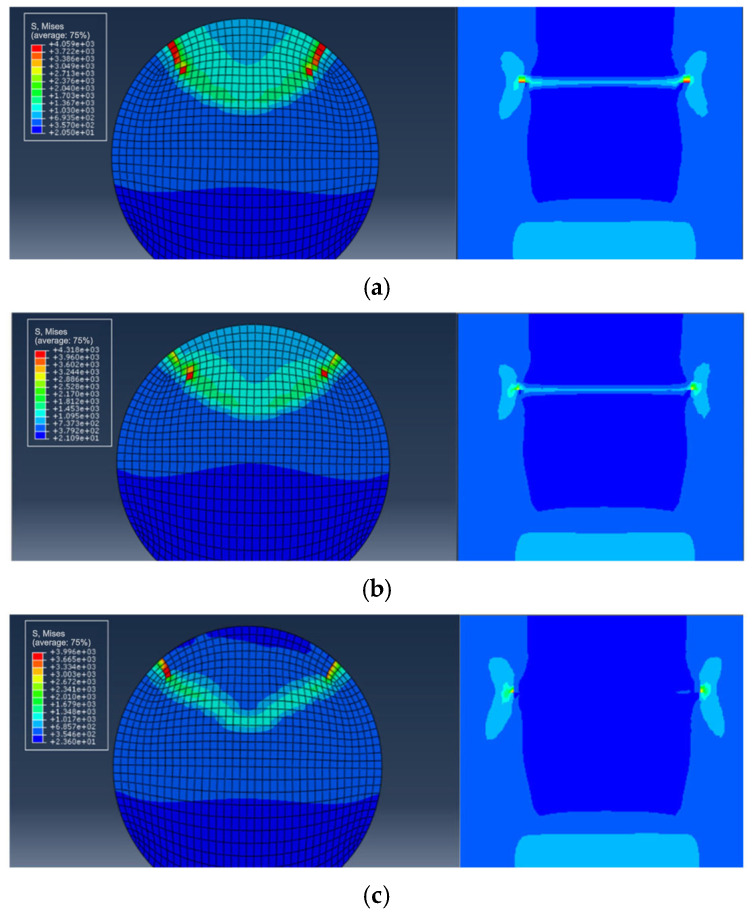
The stress state during fatigue crack propagation for steel wires with a pit and an EIFS. (**a**) *N* = 0; (**b**) *N* = 6680 cycles; (**c**) *N* = 12,989 cycles.

**Figure 16:**
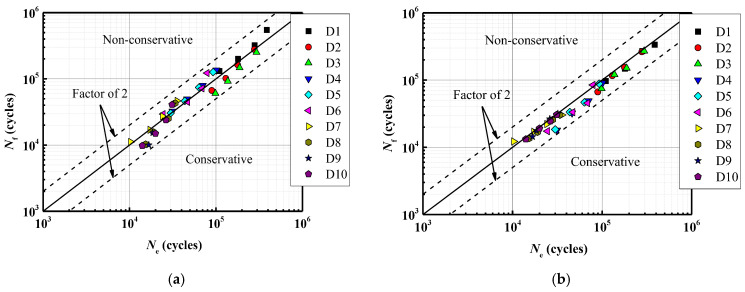
A comparison between fatigue life prediction results and test results for steel wires with a pit and an EIFS. (**a**) the maximum principal stress method. (**b**) the new method.

For the calculation example, the steel wire with double pits (No. D10-4) is selected from Table 4. The depth of both pits is 2 mm, and the EIFS is 1.9424 mm. Figure 14 reveals that the shape of the crack is semi-elliptical, resembling the assumed equivalent crack shape. The stress variation of the cross-section is similar to the single-pit case (Figure 15), and the fatigue crack initiates from the bottom of the pit and gradually propagates to both ends until it reaches the critical failure depth. The fatigue life prediction results for steel wires with double pits based on the maximum principal stress method and the new method are shown in Figure 16. All the predicted outcomes fall within a range of twice the margin of error. This equivalent approach enables the estimation of fatigue life for double-pitted steel wires, exhibiting satisfactory agreement with experimental outcomes. Therefore, this novel method holds promising potential for practical applications in the field of fatigue analysis containing multiple notches.

## 6. Conclusions

In this paper, experimental and numerical investigations on high-strength steel wires with multiple pits were carried out, and the following conclusions were obtained:

(1) The maximum principal stress range and the fatigue life in logarithmic coordinates show a good linear relationship for steel wires with various pit types. It is known that the inverse slope of the S-N curve is 4. Pits with greater depths exhibit higher susceptibility to fatigue crack formation due to the increased stress concentration.

(2) The EIFS is influenced by both the stress range and the pit depth. With the increase in the stress range and the pit depth, the EIFS also increases. It is observed that an increase in the stress range of 60 MPa corresponds to an EIFS increase of approximately 0.2 mm. It is noteworthy that the depth of the deeper pit has a more significant impact on the EIFS compared to the shallower pit.

(3) The predicted outcomes obtained using both the maximum principal stress method and the new method fall within a range of twice the margin of error. However, it should be noted that the maximum principal stress method tends to be more non-conservative. On the other hand, the new method, which combines the XFEM and the EIFS, has been validated by using experimental data and demonstrates good agreement.

## Figures and Tables

**Table 1:** Pit types and sizes.

Code	Pit Size (mm)	Pit Types
S1	*d* = 0.5, *l* = 2.65, *w* = 2.14	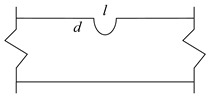 A single pit
S2	*d* = 1, *l* = 3.46, *w* = 2.94	
S3	*d* = 1.5, *l* = 3.87, *w* = 3.50	
S4	*d* = 2, *l* = 4, *w* = 4	
D1	*d*_1_ = 0.5, *l*_1_ = 2.65, *w*_1_ = 2.14; *d*_2_ = 0.5, *l*_2_ = 2.65, *w*_2_ = 2.14	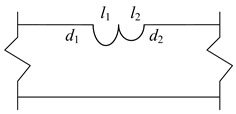 Double pits
D2	*d*_1_ = 1.0, *l*_1_ = 3.46, *w*_1_ = 2.94; *d*_2_ = 0.5, *l*_2_ = 2.65, *w*_2_ = 2.14	
D3	*d*_1_ = 1.0, *l*_1_ = 3.46, *w*_1_ = 2.94; *d*_2_ = 1.0, *l*_2_ = 3.46, *w*_2_ = 2.94	
D4	*d*_1_ = 1.5, *l*_1_ = 3.87, *w*_1_ = 3.5; *d*_2_ = 0.5, *l*_2_ = 2.65, *w*_2_ = 2.14	
D5	*d*_1_ = 1.5, *l*_1_ = 3.87, *w*_1_ = 3.5; *d*_2_ = 1, *l*_2_ = 3.46, *w*_2_ = 2.94	
D6	*d*_1_ = 1.5, *l*_1_ = 3.87, *w*_1_ = 3.50; *d*_2_ = 1.5, *l*_2_ = 3.87, *w*_2_ = 3.50	
D7	*d*_1_ = 2, *l*_1_ = 4, *w*_1_ = 4; *d*_2_ = 0.5, *l*_2_ = 2.65, *w*_2_ = 2.14	
D8	*d*_1_ = 2, *l*_1_ = 4, *w*_1_ = 4; *d*_2_ = 1, *l*_2_ = 3.46, *w*_2_ = 2.94	
D9	*d*_1_ = 2, *l*_1_ = 4, *w*_1_ = 4; *d*_2_ = 1.5, *l*_2_ = 3.87, *w*_2_ = 3.5	
D10	*d*_1_ = 2, *l*_1_ = 4, *w*_1_ = 4; *d*_2_ = 2, *l*_2_ = 4, *w*_2_ = 4	

**Table 2:** Loading parameters.

No.	Nominal Stress Range (MPa)	Stress Ratio	Maximum Load (kN)	Minimum Load (kN)
1	420	0.1	18.0	1.8
2	480		20.5	2.0
3	540		23.1	2.3
4	600		25.7	2.6

**Table 3:** EIFS values for steel wires with a single pit under different stress ranges.

Code	Pit Depth (mm)	Stress Range (MPa)	EIFS (mm)
S1-1	0.5	420	0.1030
S1-2	0.5	480	0.2550
S1-3	0.5	540	0.6839
S1-4	0.5	600	1.3521
S2-1	1.0	420	0.2596
S2-2	1.0	480	0.5589
S2-3	1.0	540	1.3897
S2-4	1.0	600	1.4458
S3-1	1.5	420	1.2048
S3-2	1.5	480	1.5905
S3-3	1.5	540	1.7632
S3-4	1.5	600	1.9388
S4-1	2.0	420	1.9288
S4-2	2.0	480	1.9661
S4-3	2.0	540	2.0287
S4-4	2.0	600	2.1266

**Table 4:** EIFSs for steel wires with double pits under different stress ranges.

Code	Pit Depth (mm)	Stress Range (MPa)	Equivalent Initial Flaw Size (mm)
D1-1	0.5–0.5	420	0.0647
D1-2	0.5–0.5	480	0.1426
D1-3	0.5–0.5	540	0.3597
D1-4	0.5–0.5	600	0.6639
D2-1	1.0–0.5	420	0.2784
D2-2	1.0–0.5	480	0.5021
D2-3	1.0–0.5	540	0.6407
D2-4	1.0–0.5	600	0.8423
D3-1	1.0–1.0	420	0.2333
D3-2	1.0–1.0	480	0.4562
D3-3	1.0–1.0	540	0.5938
D3-4	1.0–1.0	600	0.7585
D4-1	1.5–0.5	420	1.1252
D4-2	1.5–0.5	480	1.2611
D4-3	1.5–0.5	540	1.4419
D4-4	1.5–0.5	600	1.5891
D5-1	1.5–1.0	420	1.1922
D5-2	1.5–1.0	480	1.3347
D5-3	1.5–1.0	540	1.4776
D5-4	1.5–1.0	600	1.6152
D6-1	1.5–1.5	420	1.3072
D6-2	1.5–1.5	480	1.2779
D6-3	1.5–1.5	540	1.4293
D6-4	1.5–1.5	600	1.7174
D7-1	2.0–0.5	420	1.7659
D7-2	2.0–0.5	480	1.8542
D7-3	2.0–0.5	540	1.9208
D7-4	2.0–0.5	600	2.0344
D8-1	2.0–1.0	420	1.7881
D8-2	2.0–1.0	480	1.7898
D8-3	2.0–1.0	540	1.8859
D8-4	2.0–1.0	600	1.9120
D9-1	2.0–1.5	420	1.8110
D9-2	2.0–1.5	480	1.8227
D9-3	2.0–1.5	540	1.8943
D9-4	2.0–1.5	600	1.8816
D10-1	2.0–2.0	420	1.8240
D10-2	2.0–2.0	480	1.8180
D10-3	2.0–2.0	540	1.8666
D10-4	2.0–2.0	600	1.9424

## Data Availability

Data are contained within the article.
